# Thermo-Responsive Injectable Hydrogels Formed by Self-Assembly of Alginate-Based Heterograft Copolymers

**DOI:** 10.3390/gels9030236

**Published:** 2023-03-17

**Authors:** Konstantinos Safakas, Sofia-Falia Saravanou, Zacharoula Iatridi, Constantinos Tsitsilianis

**Affiliations:** The Department of Chemical Engineering, University of Patras, 26500 Patras, Greece

**Keywords:** alginate, LCST, PNIPAM, heterograft copolymer, hydrogel, thermo-responsive, injectability, self-healing

## Abstract

Polysaccharide-based graft copolymers bearing thermo-responsive grafting chains, exhibiting LCST, have been designed to afford thermo-responsive injectable hydrogels. The good performance of the hydrogel requires control of the critical gelation temperature, *T_gel_*. In the present article, we wish to show an alternative method to tune *T_gel_* using an alginate-based thermo-responsive gelator bearing two kinds of grafting chains (heterograft copolymer topology) of P(NIPAM_86_-*co*-NtBAM_14_) random copolymers and pure PNIPAM, differing in their lower critical solution temperature (LCST) about 10 °C. Interestingly, the *T_gel_* of the heterograft copolymer is controlled from the overall hydrophobic content, NtBAM, of both grafts, implying the formation of blended side chains in the crosslinked nanodomains of the formed network. Rheological investigation of the hydrogel showed excellent responsiveness to temperature and shear. Thus, a combination of shear-thinning and thermo-thickening effects provides the hydrogel with injectability and self-healing properties, making it a good candidate for biomedical applications.

## 1. Introduction

Three dimensional (3D) networks formed by hydrophilic polymers (gelators), bearing hydrophobic sticky chains and/or specific functionalities, of a large variety of macromolecular topologies (e.g., triblocks, stars, graft copolymers and/or terpolymers) have been designed and explored in aqueous media [1,2,3,4]. These formulations, also called hydrogels, due to their high water content, have attracted the immense attention of the scientific community thanks to their potential in a vast variety of applications [5,6,7,8]. As far as biomedical applications are concerned, namely drug delivery, tissue engineering, wound dressing, etc. [8,9,10,11,12,13,14], a specific design of the gelator is needed to meet the requirements of a given application that is among others, biocompatibility, controlled biodegradability, specific functionality, crosslinking density (porosity), reversibility, suitable mechanical properties, injectability and self-healing [8,9,10,11,12,13,14,15,16,17].

Stimuli-responsive water-soluble polymers, are polymers the chain conformation of which is abruptly changed (e.g., coil–globule transition), responding to changes in their environments such as temperature, pH, etc. This functionality has attracted the attention of polymer designers to achieve some of the aforementioned requirements (e.g., injectability) for a tailor-made gelator [1,4,5]. For instance, thermo-responsive polymers exhibiting LCST, transforming reversibly from hydrophilic (non-associative) to hydrophobic (associative) upon heating, have been incorporated as sticky building blocks to prepare thermo-responsive gelators [18,19,20]. At temperatures below LCST (e.g., room temperature), their aqueous solutions flow easily (sol state) and thus can be effortlessly injected into an environment of a higher temperature than the LCST. At the injection site (e.g., body temperature) the polymer gelator forms a 3D network (gel state), due to the association of the sticky LCST polymer blocks, forming the physical crosslinks. Poly(N-isopropyl acrylamide) (PNIPAM) is one of the most used LCST polymers for the fabrication of thermo-responsive gelators because it exhibits an LCST at about 32 °C which is above the room temperature (20–25 °C) but lower than the physiological temperature (37 °C), making it appropriate for biomedical applications [19,21,22].

Concerning the non-associative hydrophilic part of the gelator, forming the bridges between the crosslinks of the network, polysaccharides (very important biopolymers abundantly present in nature) have been widely used to design biocompatible and biodegradable gelators [23]. From a synthetic point of view, polysaccharides bear suitable functional groups (e.g., COOH, OH, NH_2_, etc.), capable of easy modification and grafting reactions, allowing to fabrication of carbohydrate-based graft copolymer gelators [24,25]. Among others, alginate has been used as the hydrophilic backbone of graft copolymers, bearing carboxyl groups that can be utilized for grafting LCST-type polymers, yielding thermo-responsive hydrogels. Alginate-based copolymers with PNIPAM grafting chains, exhibiting sol–gel transitions in the vicinity of the physiological temperature have been reported previously [26,27,28,29,30,31,32,33,34,35]. One of the critical parameters of the hydrogel properties is the sol–gel transition temperature (*T_gel_*), which affects the entire performance of the hydrogel, namely injectability, in situ gelling, and mechanical suitability at the conditions (pH, temperature, ionic strength) of the targeting site environment (e.g., physiological, tumor, wound). A facile tuning of *T_gel_* can be achieved by incorporating hydrophobic monomers into the PNIPAM side chains. As has been shown recently, in alginate-based graft copolymers, *T_gel_* decreases linearly with the content of the N-tert-butyl acrylamide (NtBAM) hydrophobic monomer of the P(NIPAM-*co*-NtBAM) random copolymer side chains which allow fine-tuning of the sol–gel transition. Moreover, the shift of the transition to lower temperatures significantly affects the hydrogel elasticity at the physiological temperature [36]. Recently, similar conclusions have been deduced from hyaluronan-based thermo-responsive graft copolymers [37].

In the present article, we wish to show an alternative method to tune *T_gel_* using an alginate-based thermo-responsive gelator of heterograft copolymer topology namely, a polymer backbone grafted by two types of different nature polymer chains [38]. In a two-pot reaction, alginate was grafted sequentially by P(NIPAM_86_-*co*-NtBAM_14_) (86/14 molar ratio) random copolymers and PNIPAM homopolymer pendant chains, displaying different LCSTs. The heterograft copolymer gelator exhibited *T_gel_* between those of the Alg-g-P(NIPAM_86_-*co*-NtBAM_14_) and Alg-g-P(NIPAM). Interestingly, the determining factor regulating *T_gel_* is the overall NtBAM content of both grafting chains. The prepared hydrogel exhibits also thermo-/shear- induced injectability and self-healing properties thanks to the excellent shear- and thermo-responsiveness and seems suitable for biomedical potential applications.

## 2. Results and Discussion

### 2.1. Synthesis and Characterization of Heterograft Copolymer

**Figure 1:**
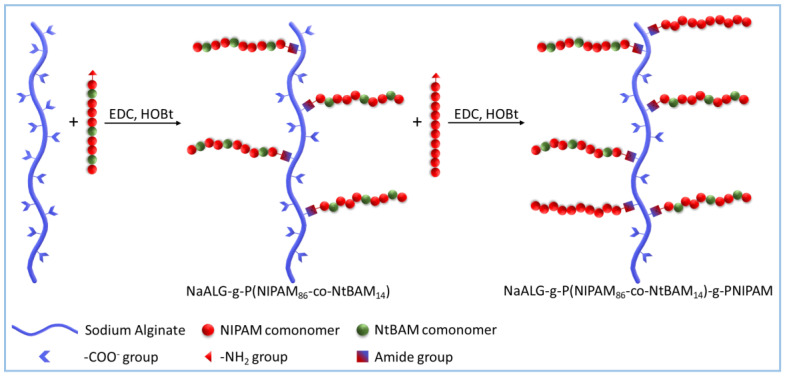
Schematic representation of the heterografting reaction. First step: synthesis of the ALG-g-P(NIPAM_86_-*co*-NtBAM_14_) graft copolymer; second step: the ALG-g-P(NIPAM_86_-*co*-NtBAM_14_) graft copolymer was grafted by the NH_2_-PNIPAM homopolymer, yielding the final ALG/HGC heterograft copolymer.

A heterograft copolymer constituted of alginate (ALG) backbone grafted by two different LCST-type thermo-responsive polymers, displaying discernible LCSTs, was synthesized according to the grafting onto methodology. Amino-functionalized NH_2_-P(NIPAM_86_-*co*-NtBAM_14_) random copolymer and NH_2_-PNIPAM homopolymer with LCST of 22 and 32 °C, respectively, were used as the grafting chains [36]. The synthesis of the copolymer was conducted In aqueous media using carbodiimide chemistry for the conjugation of the amine end-function of the grafts onto the carboxyl groups of ALG according to standard methods [31,33,34,36]. Figure 1 depicts schematically the grafting reaction, accomplished in a two-pot reaction. In the first step, the ALG-g-P(NIPAM_86_-*co*-NtBAM_14_) graft copolymer was synthesized, isolated, and characterized, as reported previously [36]. At a second step, the ALG-g-P(NIPAM_86_-*co*-NtBAM_14_) graft copolymer was grafted by the NH_2_-PNIPAM homopolymer, yielding the final ALG-[g-P(NIPAM_86_-*co*-NtBAM_14_)]-g-P(NIPAM) heterograft copolymer (ALG/HGC).

^1^H-NMR was used to characterize the ALG/HGC in terms of monomer composition, NtBAM content of the grafts, and grafting density (number of grafts per ALG backbone) (Figure S1). Table 1 shows the molecular characteristics of the ALG/HGC.

### 2.2. Thermo-Gelling Behavior

**Figure 2:**
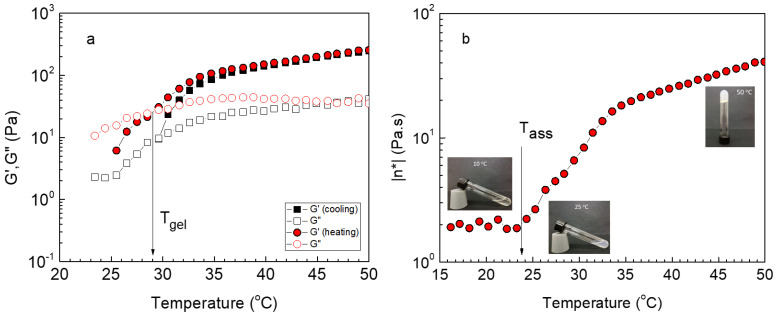
Temperature dependence of G’ (closed symbols), G” (open symbols) (**a**) and complex viscosity (heating) (**b**) at 1 Hz and strain amplitude of 0.1% for the ALG/HGC hydrogel with a cooling– heating ramp of 1 °C/min. The arrows indicate *T_gel_* in (**a**) and *T_ass_* in (**b**). The photos (inset of **b**) depict the solutions at different temperatures, showing a transparent solution at 10 °C, a turbid solution at 25 °C, and a free-standing gel at 50 °C.

Aqueous solutions of ALG/HGC, of 5 wt% polymer concentration, were prepared, and their thermo-responsive behavior was explored by using oscillatory shear experiments in the linear viscoelastic region (LVR). The pH of the solutions was adjusted to 7.4 in all cases. The formulation was charged in the rheometer at elevated temperature (gel state) and a successive cooling/heating ramp was applied with a rate of 1 °C/min, setting the frequency at 1 Hz and strain amplitude at 0.1% within the LVR. The data of this experiment, in terms of storage (G’) and loss (G”) modulus versus temperature, are demonstrated in Figure 2a. At elevated temperatures, the polymer solution behaves as gel since the storage modulus dominates to a loss modulus, whereas at low temperatures the opposite effect (G” > G’) occurs showing a sol state. The moduli follow different paths in the cooling–heating procedure, manifesting a hysteresis, due to the exchange dynamics of the associative side chains of the graft copolymer [36]. The crossover of G’/G” determines the sol/gel transition defining the *T_gel_*. As demonstrated in Figure 2 (photos), at low temperatures (e.g., 10 °C) a transparent solution is formed, which flows relatively easily (sol state). At high temperatures, well above *T_gel_* (e.g., 50 °C), a free-standing gel exists, revealing the formation of a 3D network constituted of physically crosslinked nanodomains of the associated thermo-responsive side chain, bridged by the alginate segments existing between the grafting points. The oscillatory data can also be demonstrated by plotting a single parameter (complex viscosity, η *) as a function of temperature. Figure 2b shows η * vs. T for the heating procedure. As observed, the viscosity at temperatures below 24 °C (sol state) is of the order of 2 Pa.s, which is about three orders of magnitude higher than the viscosity of the aqueous medium. Provided that the entanglement regime in aqueous polymer solutions is approximately reached at *η* = 30*η_solvent_*, [37,39] the polymer concentration of 5 wt% is clearly higher than the entanglement concentration, *C_e_*, which justifies the observed high viscosity values. More importantly, the viscosity rises above 24 °C, about one order of magnitude up to 35 °C, and continues to rise to 50 °C, but at a slower rate. Above 24 °C (defined as *T_ass_*), the solution becomes turbid (see photo at 25 °C in Figure 2b inset) revealing the onset of the hydrophobic association of the side thermo-responsive chains of the graft copolymer. Above *T_ass_* and up to *T_gel_*, the hydrophobic interactions are still very weak, as G” is still lower than G’. Above *T_gel_* these interactions are continuously strengthened, leading to the formation of a 3D network.

**Figure 3:**
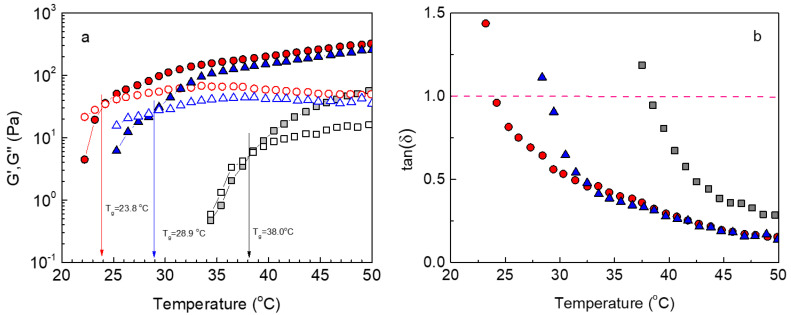
Temperature dependence of G′ (closed symbols), G″ (open symbols) (**a**), and tanδ (**b**) at 1 Hz, strain amplitude of 0.1% and heating ramp of 1 °C/min for the hydrogels formed by ALG-g-P(NIPAM_86_-*co*-NtBAM_14_) (circles), ALG/HGC (triangles) and ALG-g-P(NIPAM) (squares) gelators. The arrows in (**a**) indicate *T_gel_* and the dashed line in (**b**) denotes the sol/gel point (tanδ = 1).

Provided that the graft copolymer bears two kinds of side chains of different critical solution temperatures, the rising question is whether the P(NIPAM_86_-*co*-NtBAM_14_) chains, with the lower LCST, will associate first, followed by PNIPAM association at a higher temperature. To answer this question, we have plotted the oscillatory temperature ramp (heating procedure) for the heterografted ALG/HGC and the corresponding ALG-g-P(NIPAM_86_-*co*-NtBAM_14_), ALG-g-P(NIPAM) homo grafted copolymers. As can be seen in Figure 3, the overall behavior of the three copolymers is similar. That is, the moduli increase with temperature passing a single crossover point (G′ = G″, tanδ = 1). The main observation is that the critical temperature *T_gel_* of the ALG/HGC has been shifted to a higher temperature with respect to that of the ALG-g-P(NIPAM_86_-*co*-NtBAM_14_) precursor. This behavior reminisces the shift of *T_gel_* with varying the hydrophilic/hydrophobic content of the grafting chains observed recently [36].

**Figure 4:**
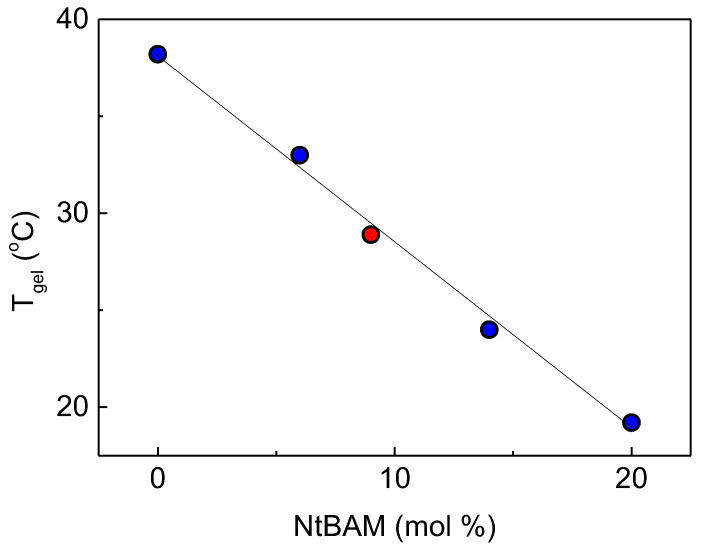
*T_gel_* as a function of the overall NtBAM content. The blue data have been taken from [36] and the red one concerns the ALG/HGC. The line is the linear regression of the data.

If we consider that the addition of the PNIPAM side chains decreases the overall NtBAM content of the side chains from 86/14 to 91/9, then the data of the heterograft copolymer should fit with the linear function of *T_gel_* versus NtBAM content as observed previously [36]. Indeed, this is the case as clearly observed in Figure 4, implying that the different side chains have been blended, exhibiting an intermediate *T_gel_*. This seems reasonable since in P(NIPAM_86_-*co*-NtBAM_14_) and PNIPAM side chains, NIPAM monomer repeating units are dominating and thus they are compatible, suggesting the formation of mixed crosslinking domains. This also suggests that *T_gel_* should be predicted by the formula:

*T_gel_* (HGC) = w_1_ *T_gel_* P(NIPAM_86_-*co*-NtBAM_14_) + w_2_ *T_gel_* (PNIPAM) (1)

where w_1_ and w_2_ (w_1_ + w_2_ = 1) are the weight fraction of the corresponding side chains on the ALG/HGC.

Indeed, applying Equation (1) with w_1_ = 0.60, *T_gel_* P(NIPAM_86_-*co*-NtBAM_14_) = 23.8 °C and w_2_ = 0.40, *T_gel_* (PNIPAM) = 38 °C, the calculated value, *T_gel_* (HGC) = 29,4 °C, is very close to the experimental one of 28.9 °C, verifying mixing of grafting chains in the crosslinked domains. The present results seem to be in very good agreement with those predicting *T_gel_* by blending two ABC triblock terpolymers with different C blocks namely: P(NIPAM-r-BA)-b-PDMAAm-b-P(NIPAM-r-BA) and (P(NIPAM-r-BA)-b-PDMAAm-b-PNIPAM) with PDMAAm (Poly(N,N-dimethyl acrylamide, hydrophilic)) and P(NIPAM-r-BA) NIPAM/butyl acrylate random copolymer (BA, hydrophobic) [40]. Note that the thermo-responsive C blocks are the pure PNIPAM and the random copolymer P(NIPAM-r-BA), where a portion of BA hydrophobic monomer has been incorporated in the PNIPAM block, which is relevant to our case.

### 2.3. Rheological Properties

**Figure 5:**
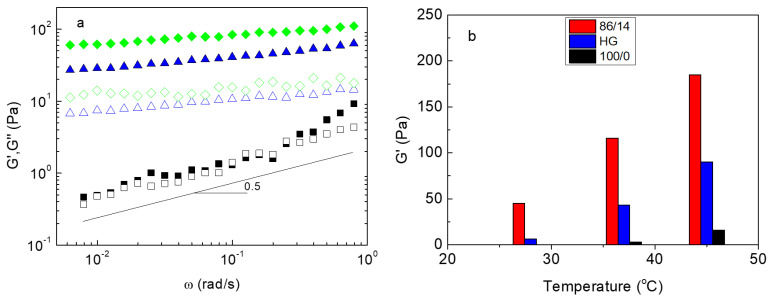
(**a**) G’ (closed symbols), G″ (open symbols) as a function of the angular frequency of 5 wt% of ALG/HGC hydrogels at various temperatures: 28 °C (black, squares), 37 °C (blue, triangles) and 45 °C (green, rhombi), the angle of the line is 0.5. (**b**) G’ at various temperatures for the hydrogels formed by ALG-g-P(NIPAM_86_-*co*-NtBAM_14_) (red bars), ALG/HGC (blue bars), and ALG-g-P(NIPAM) (black bars). The data were obtained from frequency sweeps at 1 rad/s.

The mechanical properties of the ALG/HGC formulation were further explored by oscillatory shear experiments at constant temperatures. Figure 5a demonstrates the G’, G” as a function of angular frequency at selected temperatures. At 28 °C (vicinity of *T_gel_*), tanδ (G″/G’) is close to unity and the frequency dependence power exponent is approximately 0.5, as expected for the sol–gel transition point. At the physiological temperature, 37 °C, G’ dominates G″ in the entire frequency range. The terminal zone is not visible at low frequencies, implying the formation of a 3D network with kinetically “frozen” crosslinked grafting chains, since their exchange dynamics slow down due to the increased hydrophobicity [36,41]. By increasing the temperature to 45 °C, the same behavior is observed with higher values in the moduli, suggesting further strengthening of the network.

It is interesting to look at the effect of adding PNIPAM grafts onto the ALG-g-P(NIPAM_86_-*co*-NtBAM_14_) precursor on the elasticity of the network. The storage moduli, reflecting the elasticity of the network, for the ALG/HGC and its precursor, are depicted at selected temperatures in Figure 5b. As can be seen, the storage moduli of the heterograft copolymer are lower than those of its precursor in all temperatures, although the number of grafts (stickers) per alginate backbone increased for the heterograft. This probably could be attributed to the *T_gel_* shift to higher temperatures for the ALG/HGC, which moves the gel window too, as has been previously reported [36].

**Figure 6:**
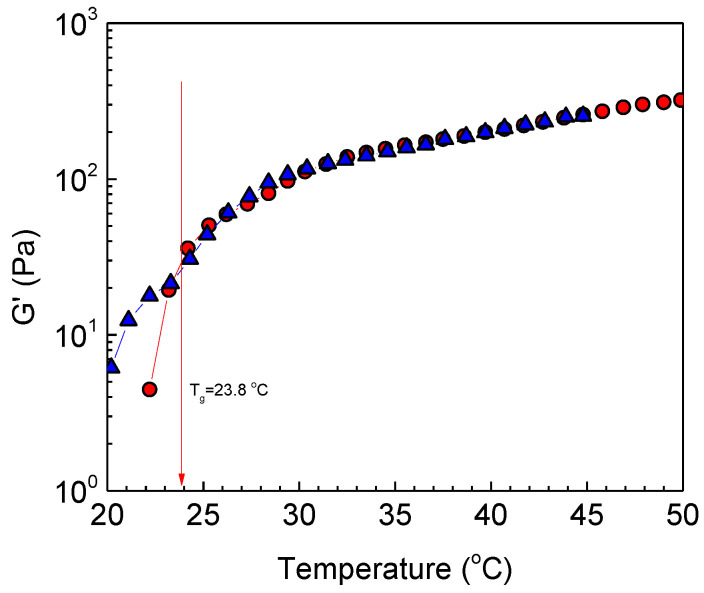
G’ versus temperature of ALG-g-P(NIPAM_86_-*co*-NtBAM_14_) (triangles) and ALG/HGC (circles). The values of the latter have been shifted in the X axis (T-ΔΤ, ΔΤ = 5.1 °C is the difference between the two *T_gels_*). The arrow indicates *T_gel_*.

Provided that the moduli increase with temperature, at a given temperature, the G’ will be lower for the formulation exhibiting higher *T_gel_* (Figure 3). To evaluate if this is valid, the G’ values of the ALG/HGC have been shifted in the temperature axis (T-ΔΤ, ΔΤ = 5.1 °C) to coincide with the *T_gel_* of its ALG-g-P(NIPAM_86_-*co*-NtBAM_14_) precursor. Indeed, the G’ values of the two formulations almost coincide above *T_gel_* (Figure 6) confirming the aforementioned hypothesis. Thus, the level of elasticity of the two grafts is almost similar above their critical *T_gel_*, implying similar crosslinking density. This is additional indirect evidence that the different side chains are blended in the crosslinking nanodomains of the network. We should mention, however, that in both systems we are in the semi-dilute regime and chain entanglements contribute to elasticity. Provided that the chain backbone in both graft copolymers is the same, we do not expect notable differences in their entanglement number density.

### 2.4. Injectability and Self-Healing

**Figure 7:**
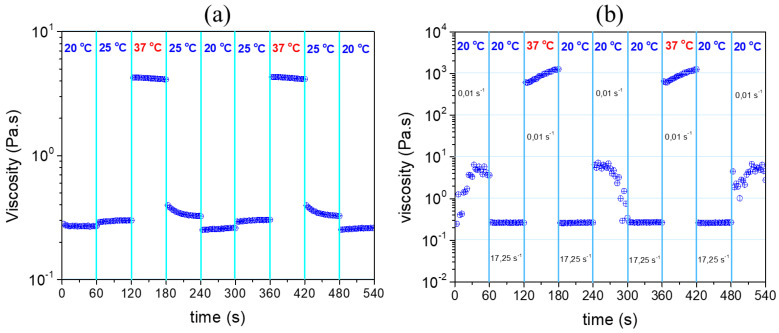
Shear viscosity stepwise time sweep in time intervals of 60 s: (**a**) altering temperatures at constant shear rate of 17.25 s^−1^ and (**b**) altering simultaneously temperature and shear rate as indicated for the ALG/HGC formulation.

As far as biomedical applications are concerned, injectability is one of the critical properties of hydrogels, as carriers of payloads. For this purpose, shear flow experiments were designed and conducted to evaluate the response of the hydrogel to temperature and shear rate. Figure 7a demonstrates consecutive shear viscosity time sweep, in time intervals of 60 s, at room (20 or 25 °C) and physiological (37 °C) temperatures. A constant shear rate at 17.25 s^−1^ was applied, which simulates an injection procedure through a 28-gauge syringe needle [41,42]. The viscosity changes slightly from 20 to 25 °C since the solution is still in the sol state and is of the order of 0.3 Pa.s which is an acceptable value for injection. Upon switching the temperature to 37 °C, the viscosity jumped to 4.2 Pa.s, more than one order of magnitude, due to the thermothickening effect (Figure 3). The viscosity of the solution responds instantly in every stepwise alteration of temperature, with very good reproducibility, showing that the formulation exhibits excellent thermo-responsiveness.

Another experiment was designed to evaluate the response of the formulation to simultaneous changes in shear and temperature, as occurs during injection from room to body temperature. Figure 7b presents the shear viscosity time sweep steps after applying shear rate and/or temperature changes. At 20 °C (room temperature), the viscosity drops instantly by changing the shear rate from 0.01 s^−1^ (approach of rest) to 17.25 s^−1^ (injection conditions). This shear thinning effect is due to the disruption of the entanglements since it is in the sol state. The viscosity increases instantly about four orders of magnitude, following the temperature jumps to 37 °C and the shear rate decrease at 0.01 s^−1^. This is mainly due to the network formation, induced by the hydrophobic association of the macromolecules at 37 °C (in situ gelling), as shown in Figure 3. This result clearly demonstrates the excellent response of the formulation to shear and temperature, implying good injectability, provided that the viscosity is low (0.3 Pa) at the injection conditions (i.e., T = 20 °C and shear rate 17.25 s^−1^). Moreover, the viscosity profiles are reproducible, irrespective of the direction of temperature changes (heating or cooling). Importantly, after the disruption of the gel at low temperature and high shear (step 6, Figure 7b) the hydrogel is instantaneously recovered at 37 °C due to the formation of the hydrophobic junctions (step 7, Figure 7b), suggesting self-healing capability of the ALG/HGC hydrogel [36,43].

**Figure 8:**
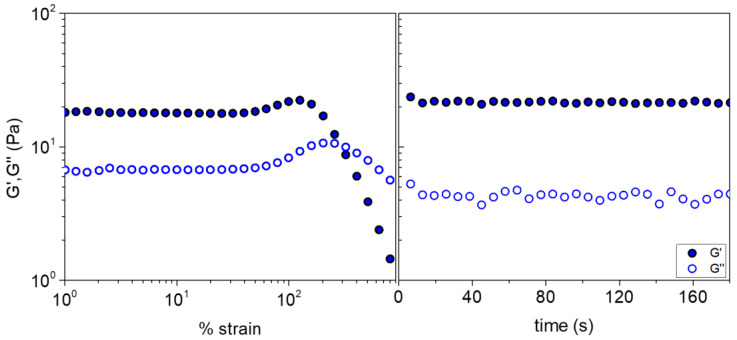
G’ (closed symbols), G″ (open symbols) as a function of strain at 1 Hz (strain sweep, **left**), followed consecutively by time sweep (**right**), applying low strain of 0.1% within the linear viscoelastic regime at 37 °C for the ALG/HGC formulation.

To further evaluate the self-healing of the formulation, an alternative experiment was designed and conducted using stepwise oscillatory tests. Particularly, the hydrogel was subjected to a strain sweep, far beyond the linear regime to disrupt the network, followed by a time sweep, setting the strain amplitude at 1%, within the linear regime at 37 °C. As seen in Figure 8, in the first step the hydrogel is liquified above 288% strain (G’/G″ crossover). The moduli decreased steadily up to 900% strain with G″ dominating G’, confirming thus the mechanical disruption of the network structure. Upon sudden decrease in the strain from 900 to 0.1%, the hydrogel is recovered instantly since G’ dominates again G″, implying the reformation of the network, confirming therefore the self-healing ability of the hydrogel.

Certainly, a large number of polymeric gelators have been designed so far to afford thermo-responsive injectable hydrogels [1,4,9,10,41,44]. In most cases, these gelators are segmented macromolecules (block copolymers) bearing associative thermo-responsive blocks in various macromolecular architectures, e.g., triblock copolymers, graft copolymers, stars, etc. The graft copolymer topology, suggested in the present case, offers some advantages with respect to synthetic copolymers (diblocks, triblocks, etc.). Beyond the synthetic simplicity and facility, the possibility to involve natural polymers such as polysaccharides, endowing the gelator with their characteristics such as biocompatibility and biodegradability, makes them compatible with biomedical applications. Particularly, the present work focuses on the issue of tunning *T_gel_*, which is critical for the hydrogel performance (e.g., injectability, 3D printability). Moreover, the heterograft type topology adopted in this work seems beneficial since it allows control of *T_gel_* and in turn of the hydrogel properties at the required conditions (room and body temperature). Importantly, it allows easy retro design by further grafting and/or adding required functionalities, depending on the specific applications. For instance, for wound healing applications, alginate-based hydrogels have been suggested. In this case, partial oxidation and dopamine functionalization are needed to promote biodegradability and bioadhesion, respectively [45].

## 3. Conclusions

A heterograft copolymer constituted of an alginate backbone bearing two kinds of grafting chains, was synthesized and explored as a gelator in aqueous media. The grafting chains are P(NIPAM_86_-*co*-NtBAM_14_) random copolymers and pure PNIPAM, differing in their LCST about 10 °C. Due to the thermo-responsive side chains, the ALG/HGC copolymer self-assembles upon heating, exhibiting sol–gel transition, *T_gel_*, at 28.9 °C, which lays between the *T_gels_* of the corresponding homo-grafted copolymers. Interestingly, the *T_gel_* of the heterograft copolymer is controlled from the overall hydrophobic content, NtBAM, of both grafts, implying the formation of blended side chains in the crosslinked nanodomains of the formed network. Rheological investigation of the hydrogel showed excellent responsiveness to temperature and shear. Thus, a combination of shear-thinning and thermo-thickening effects provides the hydrogel with injectability and self-healing properties, making it a good candidate for potential applications such as tissue engineering, and drug delivery. However, specific applications require further evaluation of additional properties such as bioadhesion, biocompatibility/toxicity, payload loading/delivery, hydrogel erosion, etc.

## 4. Materials and Methods

### 4.1. Materials

Alginic acid sodium salt (ALG) with a molecular weight range: 120,000–190,000 g/mol, and a ratio of mannuronic to guluronic units (M/G): 1.53, was purchased from Sigma-Aldrich (St. Louis, MO, USA). ALG was additionally purified by preparing a solution of 7% *w*/*v* ALG in NaOH (0.005 M), which was repetitively purified by dialysis against ultrapure water (membrane MWCO: 12,000–14,000 Da, SERVA Electrophoresis, Heidelberg, Germany). The final ALG product was obtained in its solid state through lyophilization. The synthesis of the graft copolymer ALG-g-P(NIPAM_86_-*co*-NtBAM_14_) precursor and the amino-functionalized PNIPAM side chains, that were used for the synthesis of the heterograft ALG/HGC copolymer, have been previously described [36]. N-(3-Dimethylaminopropyl)-N’-ethylcarbodiimide hydrochloride (EDC) was purchased from Alfa Aesar (Ward Hill, MA, USA) and 1-Hydroxybenzotriazole hydrate (HOBt) was obtained from Fluka (Charlotte, NC, USA). Sodium hydroxide (NaOH) was a product of Panreac (Chicago, IL, USA), and acetone and deuterated water (D_2_O) were obtained from Sigma-Aldrich (St. Louis, MO, USA). Ultrapure water was obtained by means of an ELGA Medica-R7/15 water purification device (ELGA Labwater, Woodridge, IL, USA).

### 4.2. Synthesis of the ALG/HGC Heterograft Copolymer

An amount of 2.0000 g (0.0054 mol) of ALG-g-P(NIPAM_86_-*co*-NtBAM_14_) and 0.6687 g (0.000029 mol) of NH_2_-PNIPAM was separately dissolved in 50 mL and 14 mL ultrapure water, respectively. The solutions were left under gentle stirring at 20 °C for 24 h. Afterward, the two solutions were mixed, and the mixture was left to stir at 18 °C overnight. The pH of the mixture was adjusted at pH 6 by adding NaOH (1 M). At the next step, HOBt (0.0206 g, 0.00015 mol) and EDC (0.1126 g, 0.00059 mol) were added to the mixture, and it was left under stirring at 16 °C for 24 h. The next day, the product was received by precipitation in acetone, filtered, washed with acetone, and dried in a vacuum oven at 40 °C. Then, it was dissolved in ultrapure water and further purified by dialysis against ultrapure water (membrane MWCO: 25,000 Da, SERVA Electrophoresis, Heidelberg, Germany). The final heterograft copolymer was acquired in its solid state through lyophilization.

### 4.3. Polymer Characterization

The ALG/HGC heterograft copolymer was characterized by Proton Nuclear Magnetic Resonance (^1^H-NMR), by means of a BRUKER AVANCE III HD PRODIGY ASCEND TM 600 MHz spectrometer (Billerica, MA, USA). The ALG/HGC copolymer was dissolved in D_2_O, and the ^1^H-NMR spectrum was taken at 15 °C.

### 4.4. Preparation of Polymer Solutions

An amount of 5 wt% ALG-g-P(NIPAM_86_-*co*-NtBAM_14_), ALG/HGC, and ALG-g-P(NIPAM) (the synthesis of this copolymer has been previously described [36]) aqueous solutions were prepared and left under stirring at 20 °C until homogeneity. Afterward, the solutions’ pH was set at pH 7.4, using NaOH (1 M).

### 4.5. Rheological Studies

For the rheological study of the ALG/HGC heterograft copolymer, a stress-controlled AR-2000ex rheometer with a cone–plate geometry (diameter 20 mm, angle 3°, truncation 111 μm) was used (TA Instruments, New Castle, DE, USA). The experiments were performed in the linear viscoelastic regime (determined by strain sweep tests at a frequency of 1 Hz). A Peltier system was controlling the temperature and the rheometer was equipped with a solvent trap to avoid concentration changes owing to water evaporation.

## Figures and Tables

**Table 1:** Molecular characteristics of ALG/HG copolymer.

Grafting Chain	Mn ^a^ (g/mol)	NIPAM/NtBAM Molar Ratio ^b^	Tcp ^c^ (°C)
NH_2_-PNIPAM	22,700	100/0	32
NH_2_-P(NIPAM_86_-*co*-NtBAM_14_)	17,000	86/14	22
Graft Copolymer	Mw ^d^ (×10^3^ g/mol)	Number of P(NIPAM_86_-*co*-NtBAM_14_) chains per ALG backbone ^e^	
ALG-g-P(NIPAM_86_-*co*-NtBAM_14_)	222	3.5	
ALG/HGC	262	91/9 ^f^	
		Average number of P(NIPAM_86_-*co*-NtBAM_14_) chains per ALG backbone ^e^	Average number of PNIPAM chains per ALG backbone ^e^
		3.5	1.8

^a^ From acid–base titration; ^b^ from ^1^H-NMR; ^c^ from turbidimetry, defined at the onset of the optical density abrupt increase; ^d^ calculated from the alginate Mw = 140,000 g/mol and its% weight composition from ^1^H-NMR; ^e^ from ^1^H-NMR; ^f^ total molar ratio.

## Data Availability

Not applicable.

## Supplementary Materials

The following supporting information can be downloaded at: https://www.mdpi.com/article/10.3390/gels9030236/s1, Figure S1: ^1^H-NMR spectrum of ALG/HGC in D_2_O.
