# Supplementary material for: Thermo-Responsive Injectable Hydrogels Formed by Self-Assembly of Alginate-Based Heterograft Copolymers
Source: Gels. 2023 Mar 17;9(3):236. doi: 10.3390/gels9030236 (PMC10048633; doi:10.3390/gels9030236)
Supplement: Supplementary file 1 [file gels-09-00236-s001.zip › gels-2275848-supplementary.pdf]

Supporting Information

Thermo-responsive Injectable Hydrogels Formed by Self-assembly of Alginate-based Heterograft Copolymers

Konstantinos Safakas[1], Sofia-Falia Saravanou[1], Zacharoula Iatridi[1], Constantinos Tsitsilianis[1,*]

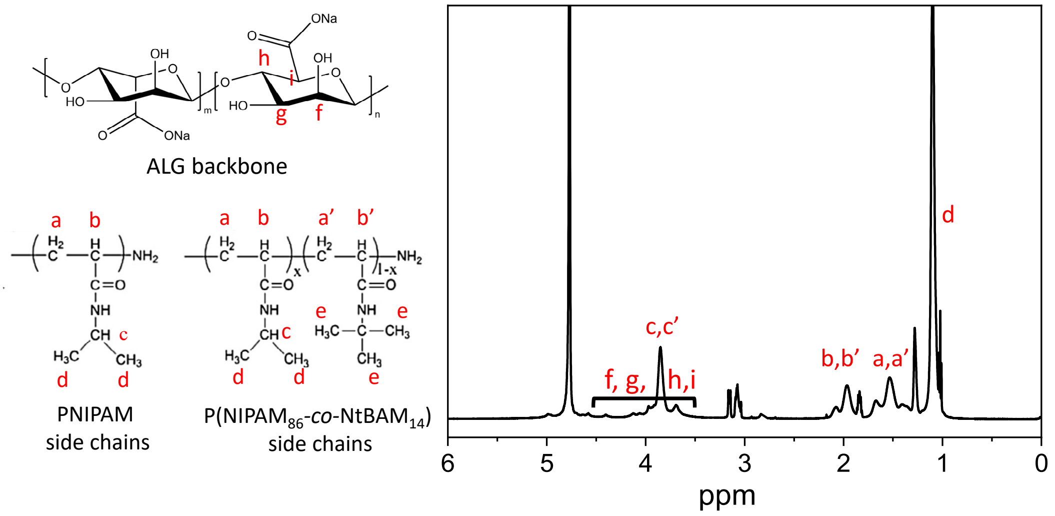

Figure S1. ^{1}H-NMR spectrum of ALG/HGC in D_2O.
